# The Role of CEUS in the Evaluation of Thyroid Cancer: From Diagnosis to Local Staging

**DOI:** 10.3390/jcm10194559

**Published:** 2021-09-30

**Authors:** Salvatore Sorrenti, Vincenzo Dolcetti, Daniele Fresilli, Giovanni Del Gaudio, Patrizia Pacini, Pintong Huang, Chiara Camponovo, Andrea Leoncini, Vito D’Andrea, Daniele Pironi, Fabrizio Frattaroli, Pierpaolo Trimboli, Maija Radzina, Vito Cantisani

**Affiliations:** 1 Department of Surgical Sciences, Faculty of Medicine, Sapienza University of Rome, Piazzale Aldo Moro 5, 00185 Rome, Italy; salvatore.sorrenti@uniroma1.it (S.S.); vito.dandrea@uniroma1.it (V.D.); daniele.pironi@uniroma1.it (D.P.); 2 Department of Radiological, Oncological, and Pathological Sciences, Faculty of Medicine, Sapienza University of Rome, Piazzale Aldo Moro 5, 00185 Rome, Italy; vincenzodolcetti@gmail.com (V.D.); daniele.fresilli@hotmail.it (D.F.); g.d.gaudio@gmail.com (G.D.G.); patry.shepsut91@gmail.com (P.P.); 3 Department of Ultrasound in Medicine, The Second Affiliated Hospital of Zhejiang University, School of Medicine, Zhejiang University, Hangzhou 310009, China; huangpintong@zju.edu.cn; 4 Research Center of Ultrasound in Medicine and Biomedical Engineering, The Second Affiliated Hospital of Zhejiang University School of Medicine, Zhejiang University, Hangzhou 310009, China; 5 Clinic for Endocrinology and Diabetology, Lugano Regional Hospital, Ente Ospedaliero Cantonale (EOC), 6900 Lugano, Switzerland; Chiara.Camponovo@eoc.ch (C.C.); Pierpaolo.Trimboli@eoc.ch (P.T.); 6 Servizio di Radiologia e Radiologia Interventistica, Istituto di Imaging della Svizzera Italiana (IIMSI), Ente Ospedaliero Cantonale (EOC), 6900 Lugano, Switzerland; andrea.leoncini@eoc.ch; 7 Department of Surgery “P. Stefanini”, Faculty of Medicine, Sapienza University of Rome, Piazzale Aldo Moro 5, 00185 Rome, Italy; fabrizio.frattaroli@uniroma1.it; 8 Faculty of Biomedical Sciences, Università della Svizzera Italiana (USI), 6900 Lugano, Switzerland; 9 Radiology Research Laboratory, Riga Stradins University, LV-1007 Riga, Latvia; mradzina@gmail.com; 10 Medical Faculty, University of Latvia; Diagnostic Radiology Institute, Paula Stradina Clinical University Hospital, LV-1007 Riga, Latvia

**Keywords:** thyroid nodule, CEUS, RF Ablation, thyroid nodule diagnosis, lymph node

## Abstract

Ultrasound often represents the first diagnostic step for thyroid nodule evaluation in clinical practice, but baseline US alone is not always effective enough to achieve thyroid nodule characterization. In the last decades new ultrasound techniques, such as CEUS, have been introduced to evaluate thyroid parenchyma as recommended by EFSUMB guidelines, for use in clinical research field, although its role is not yet clear. Several papers show the potential utility of CEUS in the differential diagnosis of benign and malignant thyroid nodules and in the analysis of lymph node involvement in neoplastic pathology. Therefore, we carried out an evaluation of the literature concerning the role of CEUS in three specific areas: the characterization of the thyroid nodule, the evaluation of minimally invasive treatment and loco-regional staging of the lymph node in proven thyroid cancer. According to evidence reported, CEUS can also play an operative role in nodular thyroid pathology as it is able to guide ablation procedures on thyroid nodule and metastatic lymph nodes, to assess the radicality of surgery, to evaluate disease relapse at the level of the margins of ablated regions and to monitor the clinical evolution of necrotic areas in immediate post-treatment setting.

## 1. Introduction

Ultrasound often represents the first diagnostic step for thyroid nodule evaluation in clinical practice. The thyroid nodule is one of the most common endocrinological disease and its incidental finding is very frequent during diagnostic neck examinations. In fact it is reported in about 10% of CT and MRI and 40–50% of ultrasound neck examinations [1,2]. It is noteworthy that the incidence of the thyroid nodule increases significantly as well as that of the malignant rate. [3]. However, baseline US alone is not always effective to achieve thyroid nodule characterization; that is why, especially in recent years, the support of other imaging modalities have become increasingly necessary to limit as much as possible the use of Fine Needle Aspiration Biopsy/Cytology, Core biopsy or even the diagnostic thyroidectomy.

CT and MRI (DWI, DCE-MRI and hybrid PET/MRI techniques) play a primary role, especially, in the visualization of deep metastatic lesions and in the evaluation of the response to treatment in non-differentiated thyroid cancer histological types [4], in the localized disease, whereas the use of Contrast-Enhanced-Ultrasonography for a second level evaluation is extensively preferable. Currently, the latest guidelines for the use of CEUS propose its employment in the evaluation of organs such as liver, kidney, testis and lymph nodes [5,6,7,8], and in the context of diagnostic procedures such as monitoring stent-graft status and in ultrasound-guided biopsies [9]. Conversely, the role of CEUS in the evaluation of nodular and diffuse thyroid pathology is not universally accepted and standardized, as it is not recommended by EFSUMB in its latest Guidelines as routine clinical practice, although it enjoys a significantly active research field [5].

The main diagnostic application of the methodology in this area of interest is represented by the characterization of the different microvascular patterns, with diagnostic accuracy superior to Color-Doppler alone [10]. Through the analysis of qualitative and quantitative parameters, CEUS is in fact able to identify pathological changes in the vascularity of both the thyroid nodule and the lymph nodes of the central and lateral compartments of the neck. CEUS is, therefore, a potentially useful tool in the differential diagnosis of benign and malignant pathologies. This is so even in the cases of nodules with indeterminate cytology, and in the analysis of lymph node involvement in neoplastic pathology. Indeed, it has been reported that increases in ultrasonographic diagnostic accuracy, especially if associated with factor BRAF V600E [11,12] or integrated with superb microvascular imaging (SMI) [13], ultrasound elastography and shear wave elastography [14].

CEUS can also play an operative role as well as a diagnostic one in nodular thyroid pathology: in fact, it can be used to guide ablation procedures on thyroid nodule and metastatic lymph nodes, to study the radicality of surgery, to evaluate the disease relapse at the level of the margins of ablated regions and to monitor the clinical evolution of necrotic areas in the immediate post-operative setting. [15,16].

Therefore, the purpose of this work was to carry out a narrative literature review on the role of CEUS regarding three specific areas: (1) characterization of the thyroid nodule; (2) evaluation of minimally invasive treatment (above all in percutaneous laser, microwave and radiofrequency ablation); (3) loco-regional staging of the lymph node in proven thyroid cancer patients.

## 2. Materials and Methods

The study was conducted mainly focusing on papers published over the last decade, as these are based on stronger scientific evidence and larger samples on which to perform retrospective studies with greater statistical significance. Research on online databases such as PubMed and Google Scholar was performed:

- to evaluate the role of CEUS in discriminating benign from malignant thyroid nodules using “CEUS or Contrast-Enhanced Ultrasonography” and “thyroid nodule or thyroid cancer” as MESH terms;
- to investigate the role of CEUS in evaluating the efficacy of treatment performed on thyroid nodules and nodal involvement the MESH terms “CEUS or Contrast-Enhanced Ultrasonography” and “thyroid nodule or thyroid cancer” and “after treatment” were used. Additionally, to evaluate the actual effectiveness of CEUS in detecting nodal metastatic involvement “CEUS or Contrast-Enhanced Ultrasonography” and “thyroid cancer lymph nodes or thyroid metastatic lymph nodes or thyroid cancer lymphatic nodes” were used as MESH terms. In this case, 118 studies were identified from January 2010, but only 80 of them were retrieved because of their true adherence to the topic.

## 3. Results and Discussion

### 3.1. CEUS r in the Diagnosis of Thyroid Nodule

Even if conventional US is recognized as the pivotal diagnostic tool to characterize thyroid nodules, some limitations, such as low reproducibility and operator-dependent performance, may reduce its diagnostic value. Furthermore, several additional US applications, including CEUS, have been reported recently in order to improve US performance in the diagnosis of thyroid nodule. In fact, CEUS actually allows us to enhance the microvascular blood flow of the nodule and then assess perfusion and vascular distribution in real time during the US examination [17]. Unfortunately, the technique has not as yet been fully standardized, there are no fixed references for quantitative or qualitative assessment and, importantly, no single CEUS parameter seems to be sensitive and specific enough for a diagnosis of malignancy. However, while the thyroid gland is rich in microvessels and after the injection of contrast agent, the parenchyma of normal thyroid exhibits rapid uniform enhancement, the vascular structures of nodules reveal enhancement in contrast with that of normal tissue [18]. These characteristics can enable the use of CEUS in the diagnosis of thyroid cancer.

Several studies showed that malignant nodules have specific CEUS enhancement patterns (i.e., heterogeneous or low enhancement) [19,20] and some relevant meta-analyses found a good level of performance for CEUS when discriminating thyroid cancer from benign lesions. One systematic review with meta-analysis [21] included articles reporting data regarding 1515 thyroid nodules with histological diagnoses only and their pre-operative CEUS evaluations. This study recorded a pooled sensitivity, specificity, positive predictive value and negative predictive value for CEUS of 85%, 82%, 83% and 85%, respectively, without inconsistency of sensitivity and with mild inconsistency of specificity [21]. A second meta-analysis, including further preliminary studies and comprehensive heterogeneity analyses carried out to assess the performance of CEUS in identifying benign and malignant thyroid nodules, confirmed that CEUS yielded high pooled sensitivity and specificity (87% and 83%) with an AUC of 0.92, indicating that it might be a tool of considerable value in the diagnosis of thyroid nodules [22]. However, there also existed a considerable heterogeneity between the studies included, which might compromise the reliability of the results [22]. This meta-analysis concluded that CEUS might provide high accuracy for the identification of thyroid nodules, but that there is still insufficient evidence that the features of CEUS can improve the diagnostic accuracy of US imaging reporting systems (such as TIRADS) at present [22]. In addition to these evidence-based data, the diagnostic value of CEUS regarding specific clusters of nodules, such as thyroid lesions with calcification, has been said to score high when selecting those in which biopsy is indicated [23]. Anyway, these results have been confirmed by all the studies [24]. It is important to note that the features of CEUS were closely related to nodule size in several studies; Yuan et al. indicated that patterns of real-time CEUS are significantly different when it comes to discerning between benign and malignant thyroid nodules, and have important clinical value [25]. Ma et al. showed that heterogeneous enhancement was an independent predictor of papillary thyroid microcarcinoma [26]. Xu et al. reported that TIRADS classification plus CEUS may be more accurate than TIRADS classification alone [27].

In addition to the above findings in literature regarding clinical applications of CEUS, some general considerations should be taken into account about its use to discriminate between thyroid cancer and benign thyroid nodules. It is well acknowledged that CEUS is associated with a rate of adverse events close to zero (1:10,000 vs. 1–12:100 of iodinated contrast agents) [5]. CEUS has a reasonable cost in many countries but is expensive in others. Only one nodule can be evaluated for each injection of contrast agent. To date, no established criterion for the patterns of enhancement and classification of thyroid nodules exists, so that it cannot be widely used worldwide [18]. Finally, CEUS is not included in any TIRADS, making it controversial in clinical practice [28].

### 3.2. The Role of CEUS in the Evaluation of Thyroid Nodules after Thermal Ablation and Radioactive Iodine Therapy

Traditionally surgery has been the main option for the treatment of thyroid nodules, however it presents several drawbacks, such as general anesthesia, scarring and the risk of induced hypothyroidism [29]. Thermal ablation has been increasingly applied in recent years to reduce the invasiveness of treatment in patients with benign thyroid nodules, recurrent thyroid cancer and metastatic cervical lymph nodes. Among the image-guided thermal ablation techniques available for solid- and mixed-structure nodules, the most commonly used are the following-laser (LA) and radiofrequency ablation (RFA) [30,31], microwave ablation [15,32,33], and the latest one introduced-high-frequency ultrasound (HIFU) [34,35]. Ultrasound (US) guided non-thermal ethanol ablation is performed in predominantly cystic nodules [36]. Some guidelines and documents expressing consensus suggest the use of image-guided thermal ablation as an alternative to surgery in patients with symptomatic thyroid nodules, more recently as first-line treatment [29,37,38]. At present, many studies have indicated a role for thermal ablation in primary thyroid microcarcinoma (PTMC) with low recurrence rate [39]. All diseases and their treatment require proper follow-up period selection, clinically relevant history data and knowledge about expected outcomes. Ultrasound is one of the most accessible methods for this purpose with all its multiparametric-spectrum advantages.

#### 3.2.1. Benign Nodules

One should be aware of the strict criteria regarding the use of thermal ablation procedures when seeking expected findings during follow-up examinations. Thyroid nodules should be symptomatic or cause mass effect and need to be confirmed as benign with at least two US-guided fine-needle aspirations (FNA) or core-needle biopsy (CNB) before treatment. A single benign diagnosis on FNA or CNB is sufficient when the nodule presents US features highly specific for benignity (isoechoic spongiform nodules or partially cystic nodules) or in the case of an autonomously functioning thyroid nodule (AFTN) at very low risk of malignancy (less than 1%) [40]. The most important indicator of the efficacy of treatment is reduction of the thyroid nodule’s volume after treatment. Reported mean data for thermal ablation varies from 50.7 to 93.5% of volume reduction [41].

Percutaneous ethanol injection (PEI) has been used for decades in thyroid nodule treatment [36] since it presents shorter procedure time and less periprocedural pain than thermal ablative procedures [42]. PEI efficacy is mainly related to proportion of solid and cystic component and is reported to be effective in the treatment of cystic nodules, especially with cystic components of >90% [43]. Reported volume reduction has been observed in 82.4–96.9% cases of cystic nodules and 65.8–86.2% in predominantly cystic nodules. This can be explained by the fact that solid components are thought to be more resistant to ethanol and that increased vascularity of nodules increases the drainage of ethanol [44].

Nevertheless, the best treatment modality for predominantly cystic thyroid nodules is still under debate because the reported recurrence rate after PEI is 26–38.3% [45], therefore, a combination of both methods may be advised for benign thyroid nodules.

Even though thermal ablation is a safe and effective procedure in predominantly solid nodules, the pattern of regrowth from margins can occur during follow-up, with a rate of 5.5% and 9% for RFA and laser ablation, respectively [46,47]. Rarely, nodules disappear completely, generally leaving scar tissue–which appears predominantly hypoechoic or has hyperechoic areas in the center of the area treated. Follow-up periods can affect study results concerning volume reduction [48]. As the size of thyroid nodules reduces gradually-mostly rapidly at the end of the first month and continues further up until at least 6–36 months. In the literature examined the primary outcome of image-guided thermal ablations was associated with a volume reduction ratio (VRR) of 60%, 66%, 62% and 53% at months 6, 12, 24 and 36. On the whole, RFA was associated with a VRR of 68%, 75% and 87%, respectively. Laser ablation was associated with a VRR of 48%, 52%, 45% and 44%, respectively. [21], suggestive of clinically significant and long-lasting volume reduction of benign thyroid nodules with some risk of regrowth (20% in the RFA and 38% in the LA) and needing lower retreatment after RFA over a 5-year follow-up period associated with a young age, large baseline volume and treatment with low-energy delivery [30]. Further promising results are also shown in the use of HIFU in a study by Trimboli et al., a reduction of at least 50% was observed at months 6, 12 and 24 in 6.4%, 16.1% and 22.5% nodules, respectively [34], while reduction of volume of 31.5% and 31.9% at 12 and 36 months, respectively, was observedin a European multicenter study [35].

US is the most widely used imaging modality for the assessment of early signs of a potential future regrowth of a nodule. Usually, the efficacy of an ablation technique is defined in terms of volume reduction >50% of the initial volume and is evaluated at one year after treatment [31,47]. Recently, some authors introduced the initial ablation ratio (IAR) as a quantitative early indicator correlated with the reduction ratio of volume during follow-up [31]. In their paper, Sim et al. evaluated the IAR identifying the ablated area on standard B-mode ultrasound images [47]. According to this study, IAR is the ratio of the ablated volume to the total volume of the nodule. If the IAR after RFA is <70%, the nodule is likely to regrow [47]. However, some limitations of US, such as low reproducibility and operator-depending performance and measuring, might reduce its accuracy in the evaluation of the ablated volume of the thyroid nodule. In particular, the ablated area can be difficult to demarcate clearly with B-mode, as it can appear as an isoechoic area compared with nonablated surrounding thyroid tissue. In these cases, a contrast-enhanced ultrasound (CEUS) is advocated after ablation to better identify the necrotic area, showing reduction in the variation of measurements and may impact on IAR definition in thyroid ablations [5].

**Figure 1:**
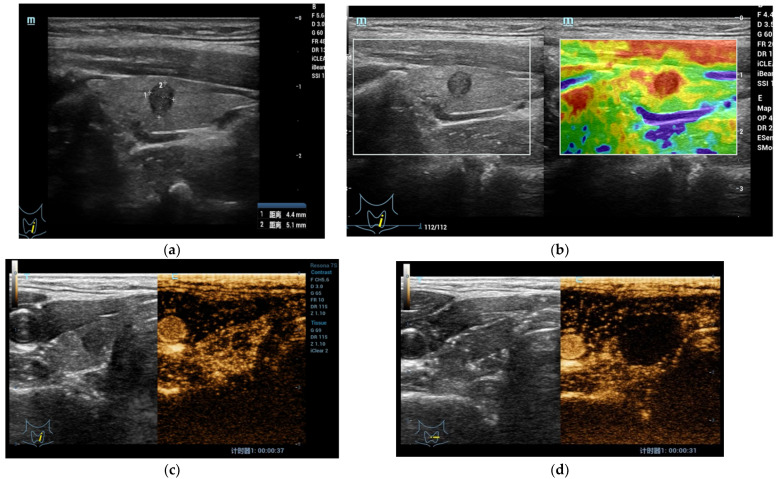
(**a**) At B-mode US, the shape of the lesion appeared taller-than-wide, hypoechoic with regular margins without internal microcalcifications (EU-TIRADS 5); (**b**) At qualitative USE evaluation, the lesion appeared stiff (completely red in the color box); (**c**) At CEUS evaluation, the lesion appeared richly vascularized similar to surrounding thyroid parenchyma without strong wash-out. At FNAC the lesion was classified as Tir 5. (**d**) After Radiofrequency Ablation, the lesion and surrounding parenchyma does not show enhancement in the CEUS mode.

CEUS is applied in some centers to precisely delineate the ablated area in thyroid nodules treated with image-guided thermal ablation (Figure 1a–d) [49,50]. In addition, US contrast agents can be directly administered to complete a standard US examination., They are safe, requires no preliminary blood testing and are well tolerated by patients. [50]. Ma et al. [51] evaluated the single-session complete ablation rate of US-guided percutaneous laser ablations for benign thyroid nodules and found that all decreased from the original size within 1 day after ablation and suggested CEUS as the main method for the evaluation of treatment efficacy. During the procedure, if CEUS shows nodules with a small amount of residual tissue at the edge, the patient requires further ablation treatment until the remnants of the lesion disappear completely. CEUS helps to clarify boundaries between viable and nonviable tissue. This might prove helpful when seeking a more precise and reproducible measure of the ablated area right after the ablation procedure and e during follow up imaging-early (3 months) and intermediate term (6 and 12 months) are the intervals suggested for follow-up with subsequent monitoring for up to 1–2 years, in order to reveal regrowth [38]. Follow-up periods can be discontinued if treated nodules disappear completely or remain as small scarring tissue [48].

#### 3.2.2. Malignant Nodules and Lymph Nodes

In cases of PTMC the retrospective studies have reported minor recurrence rates, e.g., from Yan et al. with the largest sample sizes (414 cases), the overall incidence of local tumor progression rate after RFA was only 3.62% including LNM (0.97%) and recurrence PTMC (2.42%) [39]. In addition, the patients who received additional RFA achieved good therapeutic results during follow-up. However, in a recent study, compared with PTMC, PTC (diameter > 10 mm, T1bN0M0) patients who were enrolled to undergo the TA had a relatively higher residual lesion and LNM ratio (3.03%, two in 66 cases; 1.52%, one in 66 cases, respectively) [52].

In cases of Primary Thyroid Microcarcinoma (PTMC) Zhang et al. suggested that the characteristics of high specificity, sensitivity and accuracy of CEUS might also be applied to the postoperative evaluation of PTMC and at the same time, used to access the exact ablation zone and detect the residual enhancement of suspicious lesions [53]. Even though, the postoperative pathology reports confirmed the presence of incomplete ablation in all cases where 66.7% of them presented LNM. Therefore, the authors concluded that thermal ablation should be recommended with caution as a treatment for operable patients with PTC. Of the thermal treatment methods, RFA yielded a relatively lower complete ablation rate compared with MWA and LA in recently published research. This phenomenon might be explained as follows: first, a part of the macro-calcification might not have been totally ablated, secondly, MWA is rarely affected by the heat-sink effect (local cooling of the thermal process by adjacent blood flow) that is thought to contribute to incomplete ablation and local recurrence after RFA [54]. The use of US and contrast-enhanced ultrasound (CEUS) examinations before ablation, in a study by Zhang et al. of 92 cases, confirmed by core biopsy before and after treatment, revealed that RFA can effectively eliminate low-risk PTMC with no signs of recurrence or residual tumor during follow-up periods of up to 12–18 months [55]. In a critical view of the satisfactory results of residual volume, there were great differences in the absorption rate, ranging from 10.2% to 100%, after thermal ablation in different trials [52]. PTMC is a slowly progressing disease and requires a longer and more active follow-up period to verify the efficacy of treatment [33]. Two main criteria are mandatory to evaluate ablated tissue vascularity and serum thyroglobulin (Tg) levels. Vascularity may be assessed by imaging: computed tomography [56], magnetic resonance imaging within staging protocol [57] and Color Doppler ultrasound or CEUS-loss of color signal or absence of contrast uptake within a treated lesion that was previously vascularized is adequate evidence of appropriate thermal coagulation.

The main features to be assessed in ultrasonography are: changes in nodule size, echogenicity and vascularity. Ablation areas are hypoechoic and tend to reduce in sizes. The presence of intra-nodal vascularity after RFA is an important indicator of the need to repeat the RFA procedure [58] because it should disappear in fully ablated regions. In a study a of the prognostic value of CEUS, patterns and tumor size, a comparison was made between extrathyroid extension (ETE) and non-ETE groups showing that the time from peak to one-half tumor size and wash-in slope were significantly different between the ETE and non-ETE groups [44]. Xiang et al. [59] evaluated the CEUS in the detection of neck lymph node metastasis for papillary thyroid carcinoma. The results approved heterogeneous enhancement, perfusion defects, microcalcification and centripetal/hybrid enhancement as specific criteria for malignant lymph nodes.

Hypo-enhancement and absent enhancement are considered major CEUS patterns characteristic of malignant thyroid nodules [23,60,61,62], and absent enhancement especially for thyroid tumors of 10 mm or less in diameter. The main reason that malignant thyroid tumors show a lack of blood supply is related to their complex neovascularization-once the growth becomes greater than neovascularization, tumor necrosis and embolus formation leads to hypo-enhancement on CEUS. Moreover, Zhou et al. [63] found that instead of hypo-enhancement, the nodule-to-perinodule peak intensity ratio showed the best diagnostic efficiency, with an optimal cut-off value of 0.9 [15].

In conclusion, CEUS is a precise tool before and after thyroid treatment, to use to assess the margins of recent ablation or recurrence but overlapping data between CEUS qualitative and quantitative evaluation parameters and criteria of benign and malignant features indicate a limitation in the interpretation of the nodules after treatment and create difficulties when interpreting tumor microvascularity interpretation. No single indicator is sufficiently sensitive or specific [5]. Therefore, the results should be interpreted in conjunction with clinical and case-history data, conventional US and the findings of other imaging examinations if one is to improve diagnostic accuracy in the assessment of thyroid nodules after treatment.

#### 3.2.3. Radioactive Iodine Therapy (RAI)

In patients operated for papillary thyroid carcinoma (PTC), US should be used a few months later in all patients as part of the investigation defining the response to adjuvant therapy with radioactive iodine (RAI) therapy [64,65,66]. After this first assessment, the American Thyroid Association (ATA) [65] and the European Thyroid Association (ETA) [64] only exclude the need for repeat US in patients (1) with low-risk rates, (2) with excellent response to therapy and (3) with persistently negative unstimulated Tg (u-Tg) and anti-Tg antibodies (TgAb). Even in these cases, the recommendation of repeating a US at least every 12–24 months within the first 5 years has recently been reiterated [66]. However, US frequently reveals false positive lesions that raise patient concern and required fine-needle aspiration (FNA) [67]. Most patients with PTC (except high-risk ones) will not develop disease after treatment with RAI [65]. Consequently, the detection of a neck recurrence requires that a US be performed in many patients as well as several examinations per patient [67], sometimes followed by FNA, resulting in unnecessary expenditure. These cases might be the subject of possible CEUS evaluation pre and post RAI.

In patients with macroscopically complete tumor resection who have recently received RAI, a postoperative US (before RAI) has been shown to be a valuable procedure [64,65,66,68,69] and CEUS may bring an added value. The indication of a US could be selective in the first years after RAI when postoperative US has ruled out persistent neck disease after total thyroidectomy, e.g., Rosario et al. [70] suggests that low- or intermediate-risk patients with papillary thyroid carcinoma without persistent disease after total thyroidectomy (including postoperative US and whole-body scanning) do not require repeated US examinations during the first two years after treatment with RAI. In the following years and up until the fifth year, this imaging method can be restricted to patients with u-Tg ≥ 1 ng/mL [67] and seems to be unnecessary in patients with undetectable Tg and TgAb.

### 3.3. Evaluation of Lymph-Node Local Staging Using CEUS

A correct locoregional staging of thyroid cancer through the identification of metastatic lymph nodes (LN) is essential for proper clinical and surgical management, for the treatment plan and the prognostic evaluation. Metastases from thyroid carcinoma, especially in papillary thyroid cancer (PTC) which is the most common thyroid cancer, are found in 20–50% of all cases, even in small or occult neoplastic nodules [71]. Patients with cervical lymph node metastasis (CLNM) increase the recurrence risk of PTC, and associated PTC-related death [72]. A key role in detecting pathological lymph nodes is played by ultrasound, which is more effective than mere physical examination through the palpation of the neck. At the same time, it is very effective from a cost-benefit point of view due to its widespread diffusion and accessibility. Compared to the other techniques used for the evaluation of the lymph nodes (CT, MRI techniques and PET), it turns out to be the cheapest and least invasive. This is true also during the follow-up phase [73]. Moreover, ultrasound contrast agents can be used in patients with impaired renal function and have a lower incidence of severe allergic reactions than CT and MRI contrast agents [74].

The main B-mode sonographic features of neoplastic lymph nodes are a long-axis diameter to short-axis diameter ratio (L/S ratio) of lesser than 2, a round shape, fatty hilum loss, hyper-echogenicity and the presence of calcifications and cystic components. However, all these signs can coexist both in healthy and pathological lymph nodes or have low specificity for malignancy [74]. Furthermore, there is a large discrepancy of results among the studies that analyze the effectiveness of preoperative ultrasound in the diagnosis of CLNM. As shown in the Zhao et al. and Li et al. meta-analysis, preoperative ultrasound demonstrates an intermediate sensitivity and a good but not excellent diagnostic efficacy in the diagnosis of central and lateral CLNM of PTC [75].

Thus, nowadays, CEUS might improve ultrasound diagnostic accuracy for cervical lymph node staging after PTC diagnosis. In fact, CEUS can be useful for the characterization of focal US alterations in patients with suspicious LN metastatic involvement. Specifically, CEUS emphasizes the micro-vascularization of the lymph node, where perfusion defects are a sign of metastatic involvement: poor or absent vascularization can be identified in widespread metastatic infiltration, corresponding to large areas of necrosis [8]. CEUS might also be useful for characterizing focal cortical thickening identified on grey-scale ultrasonography. Metastatic deposits are less vascularized than the adjacent nodal parenchyma, which is more evident during the parenchymal phase due to earlier contrast washout. On the contrary, more often focal thickening in benign LN displays the same enhancement features as the adjacent nodal tissue [8]. Furthermore, to improve differentiation between benign and metastatic LN perfusion kinetics has been examined too. Benign LN shows a centrifugal progression of enhancement, while a prominent centripetal enhancement is more often observed in a metastatic LN. By analysing signal time-intensity curves and the parametric images obtained through the perfusion parameters, it has also been noted that in metastatic LN compared to non-pathological LN, the difference between peak signal intensity in hyper enhancing and hypo enhancing regions is emphasized more [76].

**Figure 2:**
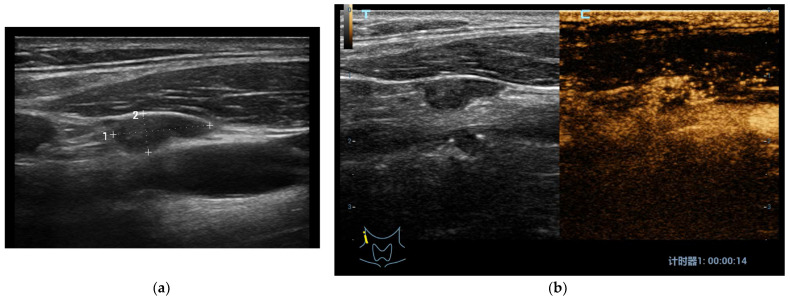
(**a**) At B-mode US in Patient with papillary thyroid carcinoma, a laterocervical node showed irregular shape, hypoechoic aspect and regular margins with no internal microcalcifications or cystic changes (low metastatic risk); (**b**) At CEUS, the laterocervical node presented rich heterogeneous and centripetal vascularization (high metastatic risk). The histological examination confirmed that it was a metastatic node of a papillary thyroid cancer.

In most of these studies the conventional US and CEUS combination is compared to histological examinations (by dissection, gun biopsy) or FNA cytology as gold standard. Hong et al. found some US and CEUS parameters useful in differential diagnosis between benign vs. metastatic LN with high specificity and statistical significance. In particular: L/S ratio < 2, ill-defined margins, hyper-echogenicity, cystic necrosis, calcification and peripheral vascularity are found at baseline US; meanwhile, centripetal or asynchronous perfusion (Figure 2a,b), non- or hyper-enhancement, perfusion defects and ring enhancing margins are found using CEUS; lymph nodes with one or more of the previous features are considered metastatic [77].

The six largest studies regarding differentiation between benign and metastatic cervical lymp nodes, conducted between 2014 and 2019, show that CEUS has a good diagnostic accuracy superior to standard US and Color-Doppler grayscale alone [59,74,77,78,79] (Table 1).

Furthermore, both Hong et al. and Chen et al. demonstrate how a combination of US and CEUS is more accurate on the whole than any of these two techniques used individually: the Hong et al. paper reported a detected a B mode US + CEUS accuracy of 92.2% (vs. 89.3% of CEUS and 84.6% of grayscale US alone) [77]; the Chen et al. paper reported a B mode US + CEUS accuracy of 92.7% (vs. 89.1% of CEUS and 80.0% of grayscale US alone) [79].

In addition, Zhan et al. proved that homogeneity, cystic change or calcification and above, all peak-time intensity, were the three strongest independent predictors for CLNM using CEUS [74]. However, only one previous study showed that no single conventional ultrasonography or CEUS characteristics were conclusive enough to distinguish metastatic thyroid nodules from indolent ones; anyway iso- or hypo-enhancements at peak time, especially in combination with several other parameters, might still be good predictors for CLNM prognoses in PTC patients [81].

In conclusion, although the studies published to date are still too few, besides being based on limited sample populations and the sensitivity and specificity values reported are quite inhomogeneous, it is possible to state that CEUS could play a role in loco-regional lymph-node evaluation in patients with malignant thyroid nodule.

## Figures and Tables

**Table 1:** Comparison of the papers based on the number of patients included in each single study and on the number of patients with at least one malignant nodule detected using CEUS; the values for sensitivity, specificity, PPV and NPV were obtained though comparison with the histological examination.

Authors of the Studies	Total Patients	Patients ± Total Patients	Sensitivity (%)	Specificity (%)	PPV ^1^	NPV ^2^	Accuracy
Xiang et al. [59]	82	65/82	82%	65%	90%	48%	79%
Zhan et al. [74]	56	33/56	65%	100%	100%	63%	78%
Hong et al. [77]	573	253/573	85%	94%	94%	86%	89%
Wang et al. [78]	285	102/285	67%	64–85% ^3^	-	-	-
Chen et al. [79]	206	46/206	90%	89%	90%	86%	89%
Tao et al. ^4^ [80]	275	127/275	72%	74%	70%	75%	73%

^1^ Positive Predictive Value; ^2^ Negative Predictive Value; ^3^ The study divides patients into two groups, PTC (>10 mm), PTMC (<10 mm); ^4^ The evaluation is based on a prediction model combining both the parameters obtained from the CEUS and clinical parameters.

## References

[1] Nachiappan A.C., Metwalli Z.A., Hailey B.S., Patel R.A., Ostrowski M.L., Wynne D.M. (2014). The Thyroid: Review of Imaging Features and Biopsy Techniques with Radiologic-Pathologic Correlation. RadioGraphics.

[2] Xie C., Cox P., Taylor N., LaPorte S. (2016). Ultrasonography of thyroid nodules: A pictorial review. Insights Imaging.

[3] Mohorea I.S., Socea B., Şerban D., Ceausu Z., Tulin A., Melinte V., Ceausu M. (2021). Incidence of thyroid carcinomas in an extended retrospective study of 526 autopsies. Exp. Ther. Med..

[4] Kushchayev S.V., Kushchayeva Y.S., Tella S.H., Glushko T., Pacak K., Teytelboym O.M. (2019). Medullary Thyroid Carcinoma: An Update on Imaging. J. Thyroid Res..

[5] Sidhu P.S., Cantisani V., Dietrich C.F., Gilja O.H., Saftoiu A., Bartels E., Bertolotto M., Calliada F., Clevert D.A., Cosgrove D. (2018). The EFSUMB Guidelines and Recommendations for the Clinical Practice of Contrast-Enhanced Ultrasound (CEUS) in Non-Hepatic Applications: Update 2017 (Long Version). Ultraschall Med..

[6] Dietrich C.F., Nolsøe C.P., Barr R.G., Berzigotti A., Burns P.N., Cantisani V., Chammas M.C., Chaubal N., Choi B.I., Clevert D.A. (2020). Guidelines and Good Clinical Practice Recommendations for Contrast-Enhanced Ultrasound (CEUS) in the Liver-Update 2020 WFUMB in Cooperation with EFSUMB, AFSUMB, AIUM, and FLAUS. Ultrasound Med. Biol..

[7] Ricci P., Laghi A., Cantisani V., Paolantonio P., Pacella S., Pagliara E., Arduini F., Pasqualini V., Trippa F., Filpo M. (2005). Contrast-enhanced sonography with SonoVue: Enhancement patterns of benign focal liver lesions and correlation with dynamic gadobenate dimeglumine-enhanced MRI. AJR Am. J. Roentgenol..

[8] Cantisani V., Bertolotto M., Weskott H.P., Romanini L., Grazhdani H., Passamonti M., Drudi F.M., Malpassini F., Isidori A., Meloni F.M. (2015). Growing indications for CEUS: The kidney, testis, lymph nodes, thyroid, prostate, and small bowel. Eur. J. Radiol..

[9] Cantisani V., Grazhdani H., Clevert D.A., Iezzi R., Aiani L., Martegani A., Fanelli F., Di Marzo L., Wlderk A., Cirelli C. (2015). EVAR: Benefits of CEUS for monitoring stent-graft status. Eur. J. Radiol..

[10] Cantisani V., Consorti F., Guerrisi A., Guerrisi I., Ricci P., Di Segni M., Mancuso E., Scardella L., Milazzo F., D’Ambrosio F. (2013). Prospective comparative evaluation of quantitative-elastosonography (Q-elastography) and contrast-enhanced ultrasound for the evaluation of thyroid nodules: Preliminary experience. Eur. J. Radiol..

[11] Zhan J., Zhang L.H., Yu Q., Li C.L., Chen Y., Wang W.P., Ding H. (2020). Prediction of cervical lymph node metastasis with contrast-enhanced ultrasound and association between presence of BRAFV600E and extrathyroidal extension in papillary thyroid carcinoma. Ther. Adv. Med. Oncol..

[12] Zhu X., Peng X., Zhu L., Xie L., Cheng F., Zhou B. (2021). Evaluation of the diagnostic performance of contrast-enhanced ultrasound combined with BRAF V600E gene detection in nodules of unclear significance by thyroid fine-needle aspiration. Gland Surg..

[13] Lu R., Meng Y., Zhang Y., Zhao W., Wang X., Jin M., Guo R. (2017). Superb microvascular imaging (SMI) compared with conventional ultrasound for evaluating thyroid nodules. BMC Med. Imaging.

[14] Gay S., Schiaffino S., Santamorena G., Massa B., Ansaldo G., Turtulici G., Giusti M. (2018). Thyroid Team at the Policlinico San Martino, Genoa. Role of Strain Elastography and Shear-Wave Elastography in a Mul-tiparametric Clinical Approach to Indeterminate Cytology Thyroid Nodules. Med. Sci. Monit..

[15] Zhang L., Zhou W., Zhan W., Peng Y., Jiang S., Xu S. (2018). Percutaneous Laser Ablation of Unifocal Papillary Thyroid Microcarcinoma: Utility of Conventional Ultrasound and Contrast-Enhanced Ultrasound in Assessing Local Therapeutic Response. World J. Surg..

[16] Zhang L., Zhou W., Zhan W. (2018). Role of ultrasound in the assessment of percutaneous laser ablation of cervical metastatic lymph nodes from thyroid carcinoma. Acta Radiol..

[17] Greis C. (2011). Quantitative evaluation of microvascular blood flow by contrast-enhanced ultrasound (CEUS). Clin. Hemorheol. Microcirc..

[18] Giusti M., Orlandi D., Melle G., Massa B., Silvestri E., Minuto F., Turtulici G. (2013). Is there a real diagnostic impact of elastosonography and contrast-enhanced ultrasonography in the management of thyroid nodules?. J. Zhejiang Univ. Sci. B.

[19] Zhang Y., Zhou P., Tian S.M., Zhao Y.F., Li J.L., Li L. (2017). Usefulness of combined use of contrast-enhanced ultrasound and TI-RADS classification for the differentiation of benign from malignant lesions of thyroid nodules. Eur. Radiol..

[20] Wang Y., Nie F., Liu T., Yang D., Li Q., Li J., Song A. (2018). Revised Value of Contrast-Enhanced Ultrasound for Solid Hypo-Echoic Thyroid Nodules Graded with the Thyroid Imaging Reporting and Data System. Ultrasound Med. Biol..

[21] Trimboli P., Castellana M., Virili C., Havre R.F., Bini F., Marinozzi F., D’Ambrosio F., Giorgino F., Giovanella L., Prosch H. (2020). Performance of contrast-enhanced ultrasound (CEUS) in assessing thyroid nodules: A systematic review and meta-analysis using histological standard of reference. Radiol. Med..

[22] Zhang J., Zhang X., Meng Y., Chen Y. (2020). Contrast-enhanced ultrasound for the differential diagnosis of thyroid nodules: An updated meta-analysis with comprehensive heterogeneity analysis. PLoS ONE.

[23] Jiang J., Shang X., Wang H., Xu Y.B., Gao Y., Zhou Q. (2015). Diagnostic value of contrast-enhanced ultrasound in thyroid nodules with calcification. Kaohsiung J. Med. Sci..

[24] Friedrich-Rust M., Sperber A., Holzer K., Diener J., Grünwald F., Badenhoop K., Weber S., Kriener S., Herrmann E., Bechstein W.O. (2010). Real-time elastography and contrast-enhanced ultrasound for the assessment of thyroid nodules. Exp. Clin. Endocrinol. Diabetes.

[25] Yuan Z., Quan J., Yunxiao Z., Jian C., Zhu H. (2015). Contrast-enhanced ultrasound in the diagnosis of solitary thyroid nodules. J. Cancer Res. Ther..

[26] Ma H.J., Yang J.C., Leng Z.P., Chang Y., Kang H., Teng L.H. (2017). Preoperative prediction of papillary thyroid microcarcinoma via multiparameter ultrasound. Acta Radiol..

[27] Xu Y., Qi X., Zhao X., Ren W., Ding W. (2019). Clinical diagnostic value of contrast-enhanced ultrasound and TI-RADS classification for benign and malignant thyroid tumors: One comparative cohort study. Medicine.

[28] Durante C., Grani G., Lamartina L., Filetti S., Mandel S.J., Cooper D.S. (2018). The Diagnosis and Management of Thyroid Nodules: A Review. JAMA.

[29] Kim J., Baek J.H., Lim H.K., Ahn H.S., Baek S.M., Choi Y.J., Choi Y.J., Chung S.R., Ha E.J., Hahn S.Y. (2018). 2017 Thyroid Radiofrequency Ablation Guideline: Korean Society of Thyroid Radiology. Korean J. Radiol..

[30] Bernardi S., Giudici F., Cesareo R., Antonelli G., Cavallaro M., Deandrea M., Giusti M., Mormile A., Negro R., Palermo A. (2020). Five-Year Results of Radiofrequency and Laser Ablation of Benign Thyroid Nodules: A Multicenter Study from the Italian Minimally Invasive Treatments of the Thyroid Group. Thyroid.

[31] Mauri G., Gennaro N., Lee M.K., Baek J.H. (2019). Laser and Radiofrequency Ablations for Benign and Malignant Thyroid Tumors. Int. J. Hyperth..

[32] Yan J., Qiu T., Lu J., Wu Y., Yang Y. (2018). Microwave ablation induces a lower systemic stress response in patients than open surgery for treatment of benign thyroid nodules. Int. J. Hyperth..

[33] Teng D.K., Li W.H., Du J.R., Wang H., Yang D.Y., Wu X.L. (2020). Effects of Microwave Ablation on Papillary Thyroid Microcarcinoma: A Five-Year Follow-Up Report. Thyroid.

[34] Trimboli P., Pelloni F., Bini F., Marinozzi F., Giovanella L. (2019). High-intensity focused ultrasound (HIFU) for benign thyroid nodules: 2-year follow-up results. Endocrine.

[35] Monpeyssen H., Ben Hamou A., Hegedüs L., Ghanassia É., Juttet P., Persichetti A., Bizzarri G., Bianchini A., Guglielmi R., Raggiunti B. (2020). High-intensity focused ultrasound (HIFU) therapy for benign thyroid nodules: A 3-year retrospective multicenter follow-up study. Int. J. Hyperth..

[36] Bernardi S., Stacul F., Zecchin M., Dobrinja C., Zanconati F., Fabris B. (2016). Radiofrequency ablation for benign thyroid nodules. J. Endocrinol. Investig..

[37] Dietrich C.F., Müller T., Bojunga J., Dong Y., Mauri G., Radzina M., Dighe M., Cui X.W., Grünwald F., Schuler A. (2018). Statement and Recommendations on Interventional Ultrasound as a Thyroid Diagnostic and Treatment Procedure. Ultrasound Med. Biol..

[38] Papini E., Monpeyssen H., Frasoldati A., Hegedüs L. (2020). European Thyroid Association Clinical Practice Guideline for the Use of Image-Guided Ablation in Benign Thyroid Nodules. Eur. Thyroid J..

[39] Yan L., Lan Y., Xiao J., Lin L., Jiang B., Luo Y. (2021). Long-term outcomes of radiofrequency ablation for unifocal low-risk papillary thyroid microcarcinoma: A large cohort study of 414 patients. Eur. Radiol..

[40] Park H.S., Baek J.H., Park A.W., Chung S.R., Choi Y.J., Lee J.H. (2017). Thyroid Radiofrequency Ablation: Updates on Innovative Devices and Techniques. Korean J. Radiol..

[41] Radzina M., Cantisani V., Rauda M., Nielsen M.B., Ewertsen C., D’Ambrosio F., Prieditis P., Sorrenti S. (2017). Update on the role of ultrasound guided radiofrequency ablation for thyroid nodule treatment. Int. J. Surg..

[42] Baek J.H., Ha E.J., Choi Y.J., Sung J.Y., Kim J.K., Shong Y.K. (2015). Radiofrequency versus Ethanol Ablation for Treating Predominantly Cystic Thyroid Nodules: A Randomized Clinical Trial. Korean J. Radiol..

[43] Kim Y.J., Baek J.H., Ha E.J., Lim H.K., Lee J.H., Sung J.Y., Kim J.K., Kim T.Y., Kim W.B., Shong Y.K. (2012). Cystic versus predominantly cystic thyroid nodules: Efficacy of ethanol ablation and analysis of related factors. Eur. Radiol..

[44] Sung J.Y., Baek J.H., Kim K.S., Lee D., Yoo H., Kim J.K., Park S.H. (2013). Single-session treatment of benign cystic thyroid nodules with ethanol versus radiofrequency ablation: A prospective randomized study. Radiology.

[45] Suh C.H., Baek J.H., Ha E.J., Choi Y.J., Lee J.H., Kim J.K., Chung K.W., Kim T.Y., Kim W.B., Shong Y.K. (2015). Ethanol ablation of predominantly cystic thyroid nodules: Evaluation of recurrence rate and factors related to recurrence. Clin. Radiol..

[46] Lim H.K., Lee J.H., Ha E.J., Sung J.Y., Kim J.K., Baek J.H. (2013). Radiofrequency ablation of benign non-functioning thyroid nodules: 4-year follow-up results for 111 patients. Eur. Radiol..

[47] Sim J.S., Baek J.H., Lee J., Cho W., Jung S.I. (2017). Radiofrequency ablation of benign thyroid nodules: Depicting early sign of regrowth by calculating vital volume. Int. J. Hyperth..

[48] Baek J.H., Lee J.H., Valcavi R., Pacella C.M., Rhim H., Na D.G. (2011). Thermal ablation for benign thyroid nodules: Radiofrequency and laser. Korean J. Radiol..

[49] Cesareo R., Palermo A., Benvenuto D., Cella E., Pasqualini V., Bernardi S., Stacul F., Angeletti S., Mauri G., Ciccozzi M. (2019). Correction to: Efficacy of radiofrequency ablation in autonomous functioning thyroid nodules. A systematic review and meta-analysis. Rev. Endocr. Metab. Disord..

[50] Pacella C.M., Mauri G., Cesareo R., Paqualini V., Cianni R., De Feo P., Gambelunghe G., Raggiunti B., Tina D., Deandrea M. (2017). A comparison of laser with radiofrequency ablation for the treatment of benign thyroid nodules: A propensity score matching analysis. Int. J. Hyperth..

[51] Ma S., Zhou P., Wu X., Tian S., Zhao Y. (2016). Detection of the Single-Session Complete Ablation Rate by Contrast-Enhanced Ultrasound during Ultrasound-Guided Laser Ablation for Benign Thyroid Nodules: A Prospective Study. Biomed Res. Int..

[52] Min Y., Wang X., Chen H., Chen J., Xiang K., Yin G. (2020). Thermal Ablation for Papillary Thyroid Microcarcinoma: How Far We Have Come?. Cancer Manag. Res..

[53] Zhang M., Tufano R.P., Russell J.O., Zhang Y., Zhang Y., Qiao Z., Luo Y. (2020). Ultrasound-Guided Radiofrequency Ablation Versus Surgery for Low-Risk Papillary Thyroid Microcarcinoma: Results of Over 5 Years’ Follow-Up. Thyroid.

[54] Yue W., Wang S., Yu S., Wang B. (2014). Ultrasound-guided percutaneous microwave ablation of solitary T1N0M0 papillary thyroid microcarcinoma: Initial experience. Int. J. Hyperth..

[55] Zhang M., Luo Y., Zhang Y., Tang J. (2016). Efficacy and Safety of Ultrasound-Guided Radiofrequency Ablation for Treating Low-Risk Papillary Thyroid Microcarcinoma: A Prospective Study. Thyroid.

[56] Mitchell A.L., Gandhi A., Scott-Coombes D., Perros P. (2016). Management of thyroid cancer: United Kingdom National Multidisciplinary Guidelines. J. Laryngol. Otol..

[57] Warren Frunzac R., Richards M. (2016). Computed Tomography and Magnetic Resonance Imaging of the Thyroid and Parathyroid Glands. Front. Horm. Res..

[58] Baek J.H., Kim Y.S., Sung J.Y., Choi H., Lee J.H. (2011). Locoregional control of metastatic well-differentiated thyroid cancer by ultrasound-guided radiofrequency ablation. AJR Am. J. Roentgenol..

[59] Xiang D., Hong Y., Zhang B., Huang P., Li G., Wang P., Li Z. (2014). Contrast-enhanced ultrasound (CEUS) facilitated US in detecting lateral neck lymph node metastasis of thyroid cancer patients: Diagnosis value and enhancement patterns of malignant lymph nodes. Eur. Radiol..

[60] Schleder S., Janke M., Agha A., Schacherer D., Hornung M., Schlitt H.J., Stroszczynski C., Schreyer A.G., Jung E.M. (2015). Preoperative differentiation of thyroid adenomas and thyroid carcinomas using high resolution contrast-enhanced ultrasound (CEUS). Clin. Hemorheol. Microcirc..

[61] Zhao R.N., Zhang B., Yang X., Jiang Y.X., Lai X.J., Zhang X.Y. (2015). Logistic Regression Analysis of Contrast-Enhanced Ultrasound and Conventional Ultrasound Characteristics of Sub-centimeter Thyroid Nodules. Ultrasound Med. Biol..

[62] Li F., Luo H. (2013). Comparative study of thyroid puncture biopsy guided by contrast-enhanced ultrasonography and conventional ultrasound. Exp. Ther. Med..

[63] Zhou X., Zhou P., Hu Z., Tian S.M., Zhao Y., Liu W., Jin Q. (2018). Diagnostic Efficiency of Quantitative Contrast-Enhanced Ultrasound Indicators for Discriminating Benign from Malignant Solid Thyroid Nodules. J. Ultrasound Med..

[64] Leenhardt L., Erdogan M.F., Hegedus L., Mandel S.J., Paschke R., Rago T., Russ G. (2013). European thyroid association guidelines for cervical ultrasound scan and ultrasound-guided techniques in the postoperative management of patients with thyroid cancer. Eur. Thyroid J..

[65] Haugen B.R., Alexander E.K., Bible K.C., Doherty G.M., Mandel S.J., Nikiforov Y.E., Pacini F., Randolph G.W., Sawka A.M., Schlumberger M. (2016). 2015 American Thyroid Association Management Guidelines for Adult Patients with Thyroid Nodules and Differentiated Thyroid Cancer: The American Thyroid Association Guidelines Task Force on Thyroid Nodules and Differentiated Thyroid Cancer. Thyroid.

[66] Pacini F., Basolo F., Bellantone R., Boni G., Cannizzaro M.A., De Palma M., Durante C., Elisei R., Fadda G., Frasoldati A. (2018). Italian consensus on diagnosis and treatment of differentiated thyroid cancer: Joint statements of six Italian societies. J. Endocrinol. Investig..

[67] Verburg F.A., Mäder U., Giovanella L., Luster M., Reiners C. (2018). Low or Undetectable Basal Thyroglobulin Levels Obviate the Need for Neck Ultrasound in Differentiated Thyroid Cancer Patients After Total Thyroidectomy and 131I Ablation. Thyroid.

[68] Lepoutre-Lussey C., Maddah D., Golmard J.L., Russ G., Tissier F., Trésallet C., Menegaux F., Aurengo A., Leenhardt L. (2014). Post-operative neck ultrasound and risk stratification in differentiated thyroid cancer patients with initial lymph node involvement. Eur. J. Endocrinol..

[69] Matrone A., Gambale C., Piaggi P., Viola D., Giani C., Agate L., Bottici V., Bianchi F., Materazzi G., Vitti P. (2017). Postoperative Thyroglobulin and Neck Ultrasound in the Risk Restratification and Decision to Perform 131I Ablation. J. Clin. Endocrinol. Metab..

[70] Rosario P.W., Calsolari G.F.M.M.R. (2019). The risk of recurrence within the first five years is very low in patients with papillary thyroid carcinoma treated with radioiodine. Arch. Head Neck Surg..

[71] Cooper D.S., Doherty G.M., Haugen B.R., Kloos R.T., Lee S.L., Mandel S.J., Mazzaferri E.L., McIver B., Pacini F., American Thyroid Association (ATA) Guidelines Taskforce on Thyroid Nodules and Differentiated Thyroid Cancer (2009). Revised American Thyroid Association management guidelines for patients with thyroid nodules and differentiated thyroid cancer. Thyroid.

[72] Baek S.K., Jung K.Y., Kang S.M., Kwon S.Y., Woo J.S., Cho S.H., Chung E.J. (2010). Clinical risk factors associated with cervical lymph node recurrence in papillary thyroid carcinoma. Thyroid.

[73] Wang L.Y., Ganly I. (2018). Post-treatment surveillance of thyroid cancer. Eur. J. Surg. Oncol..

[74] Zhan J., Diao X.H., Chen Y., Wang W.P., Ding H. (2019). Homogeneity Parameter in Contrast-Enhanced Ultrasound Imaging Improves the Classification of Abnormal Cervical Lymph Node after Thyroidectomy in Patients with Papillary Thyroid Carcinoma. Biomed Res. Int..

[75] Zhao H., Li H. (2019). Meta-analysis of ultrasound for cervical lymph nodes in papillary thyroid cancer: Diagnosis of central and lateral compartment nodal metastases. Eur. J. Radiol..

[76] Rubaltelli L., Corradin S., Dorigo A., Tregnaghi A., Adami F., Rossi C.R., Stramare R. (2007). Automated quantitative evaluation of lymph node perfusion on contrast-enhanced sonography. AJR Am. J. Roentgenol..

[77] Hong Y.R., Luo Z.Y., Mo G.Q., Wang P., Ye Q., Huang P.T. (2017). Role of Contrast-Enhanced Ultrasound in the Pre-operative Diagnosis of Cervical Lymph Node Metastasis in Patients with Papillary Thyroid Carcinoma. Ultrasound Med. Biol..

[78] Wang Y., Nie F., Wang G., Liu T., Dong T., Sun Y. (2021). Value of Combining Clinical Factors, Conventional Ultrasound, and Contrast-Enhanced Ultrasound Features in Preoperative Prediction of Central Lymph Node Metastases of Different Sized Papillary Thyroid Carcinomas. Cancer Manag. Res..

[79] Chen L., Chen L., Liu J., Wang B., Zhang H. (2020). Value of Qualitative and Quantitative Contrast-Enhanced Ultrasound Analysis in Preoperative Diagnosis of Cervical Lymph Node Metastasis from Papillary Thyroid Carcinoma. J. Ultrasound Med..

[80] Tao L., Zhou W., Zhan W., Li W., Wang Y., Fan J. (2020). Preoperative Prediction of Cervical Lymph Node Metastasis in Papillary Thyroid Carcinoma via Conventional and Contrast-Enhanced Ultrasound. J. Ultrasound Med..

[81] Zhan J., Diao X., Chen Y., Wang W., Ding H. (2019). Predicting cervical lymph node metastasis in patients with papillary thyroid cancer (PTC)—Why contrast-enhanced ultrasound (CEUS) was performed before thyroidectomy. Clin. Hemorheol. Microcirc..

